# Cross-ancestry analysis of gestational diabetes mellitus identifies novel loci, drug targets and biological pathways

**DOI:** 10.3389/fendo.2026.1718746

**Published:** 2026-04-23

**Authors:** Wendi Zhao, Hong Hu, Min Li, Ling Liu, Wei Peng, Yijing Chu

**Affiliations:** 1 Department of Obstetrics, Qingdao University Affiliated Hospital, Qingdao, China; 2 Nankai University Affiliated Hospital of Obstetrics and Gynecology, Tianjin, China; 3 Clinical Medicine, Nantong University, Nantong, China

**Keywords:** ATRAID, ELL2, genetics, genome-wide association study, gestational diabetes mellitus

## Abstract

**Background:**

Gestational diabetes mellitus (GDM) is a common pregnancy complication with adverse maternal and fetal outcomes, yet its genetic basis is not fully understood. Previous genome-wide association studies (GWAS) have identified only a limited number of susceptibility loci, most of which overlap with genes for type 2 diabetes (T2D).

**Methods:**

We performed a meta-analysis of GWAS datasets from British, Finnish, and Chinese populations to identify novel genetic susceptibility loci for GDM. Functional annotation of associated variants was conducted using FUMA. We assessed cross-population genetic correlations (Popcorn) and fine-mapped ancestry-specific signals (SuSiEx). Candidate gene was then prioritized using multiple approaches, including eQTL mapping, MAGMA, TWAS-Fusion, GCTA-mBAT, and PoPs. Finally, we integrated proteomic data from BLISS and performed in-depth pathway and tissue enrichment analyses using MAGMA and GSA-MiXeR.

**Results:**

Our multi-pronged approach identified two novel susceptibility genes associated with GDM risk: *ELL2* and *ATRAID*. In addition, three enriched biological pathways linked to GDM loci were discovered, including: the regulation of hexokinase activity, the regulation of insulin, and the regulation of protein.

**Conclusion:**

Our study links the expression of *ELL2* and *ATRAID* with the risk of GDM, and identifies three GDM-related enriched pathways. These findings provide new insights into the pathogenesis of GDM and highlights potential new targets for future research and therapeutic intervention.

## 1 Introduction

Affecting an estimated 14% of pregnancies globally, GDM is one of the most common metabolic disorders of pregnancy (1). It is characterized by the first onset of impaired glucose tolerance during pregnancy, and poses significant short- and long-term health risks to both the mother and child (2). However, its underlying genetic cause remains largely unknown. So far, most genetic studies have found a limited number of risk genes, with the majority overlapping with those already known associated to be associated with T2D (3, 4). Unraveling the particular genetic underpinnings of GDM is therefore critical for identifying high-risk women and developing preventive strategies.

Epidemiological studies have established that GDM has a strong genetic predisposition (5). In recent years, GWAS have become the primary method for investigating the genetic architecture of this and other complex diseases (6). However, the application of GWAS to GDM still faces challenges. GWAS analyses in diverse populations have facilitated a deeper understanding of the genetic susceptibility and pathogenesis of GDM Previous studies have successfully identified some risk loci, such as *MTNR1B*, *GCKR*, and *CDKAL1 (*7–9), but they are often susceptible to key limitations: relatively small sample sizes, highly homogenous (often single-ancestry) populations, and a lack of large-scale data from East Asian populations. Since the allele frequencies and linkage disequilibrium patterns can differ between populations, cross-ancestry studies are a powerful tool for discovering novel loci and help distinguish which genetic effects are shared globally.

To address these research gaps, we conducted the largest cross-ancestry genome-wide meta-analysis of GDM to date, integrating three large-scale GWAS datasets from East Asian (Chinese) and European (Finnish and British) populations (totaling 31,469 cases and 338,305 controls). We further applied a comprehensive functional annotation framework using multiple analytical tools, including functional mapping and annotation (FUMA), fine-mapping with SuSiE-X, colocalization analysis, eQTL mapping, nearest gene assignment, TWAS-Fusion, GCTA-mBAT, PoPs, MAGMA, pQTL analysis via BLISS, tissue-specific spatial gene expression (GSMap), and pathway enrichment analyses using MAGMA and GSA-MiXeR. Together, by systematically delineating the genetic architecture and potential pathogenic mechanisms of GDM, these approaches have the potential to identify novel, biologically relevant genetic markers, thus providing a theoretical foundation for future biological investigations and clinical translation. Additionally, distinguishing which genetic risk factors are shared across ancestries versus unique to specific populations could have important clinical implications informing more personalized risk assessment and guiding future mechanistic studies of GDM.

## 2 Materials and methods

### 2.1 Study design

**Figure 1:**
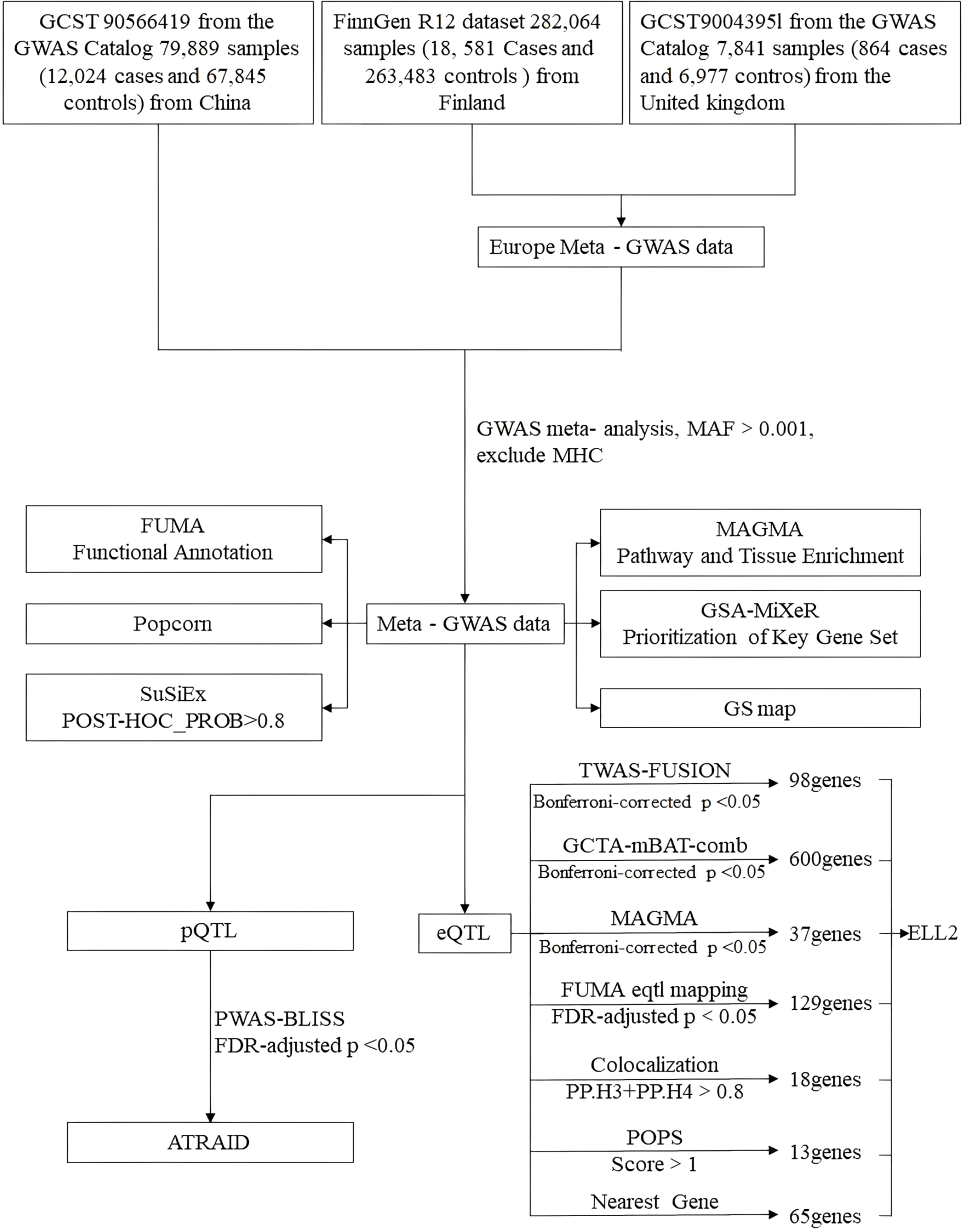
Flowchart of the study. FUMA, Functional Mapping and Annotation; FUSION, Functional summary-based imputation; GCTA, Genome-wide complex trait analysis; GWAS, Genome-wide association study; mBAT, Multivariate set-based association test; PoPS, Polygenic priority score; PWAS, Proteome-wide association study; TWAS, Transcriptome-wide association study; GS map, Genetically Informed Spatial Mapping.

A multi-stage data analysis workflow is followed, as in **Figure 1**. First, we collected publicly available GWAS summary statistics for GDM from three large-scale databases. Next, we performed a meta-analysis combining data from different ancestries to maximize statistical power. Subsequently, we identified and prioritized potential causal genes using different bioinformatic tools. Finally, we used MAGMA and GSA-MiXeR to identify biological pathways related to GDM.

### 2.2 Datasets

We integrated three large GDM GWAS datasets from different populations. The first was a Chinese cohort (GWAS Catalog GCST90566419) comprising 79,869 participants (12,024 GDM cases and 67,845 controls). GDM in this cohort was diagnosed by a standard 75 g oral glucose tolerance test (OGTT) between 24 and 28 weeks’ gestation, using the IADPSG criteria (fasting plasma glucose ≥ 5.1 mmol/L, 1-hour ≥ 10.0 mmol/L, or 2-hour ≥ 8.5 mmol/L). The second dataset was from a Finnish population (FinnGen R12), including 282,064 participants (18,581 GDM cases and 263,483 controls). GDM case status in FinnGen was identified via hospital registry records using the ICD-10 code O24.4 (gestational diabetes mellitus). The third dataset was a UK population (GWAS Catalog GCST90043951), consisting of 7,841 participants (864 cases and 6,977 controls). GDM diagnoses in the UK cohort were based on clinical records (e.g., documented OGTT results) as reported in the original study. Expression quantitative trait loci (eQTL) (gene expression) data were sourced from the GTEx v8 dataset, which provides extensive gene expression information across 49 different tissues (10). After excluding male-specific tissues, we included data from the remaining 47 tissues in the analysis. pQTL (protein expression) data were sourced from two large proteomics projects: the UK Biobank Pharma Proteomics Project (UKB-PPP), which provides 2,923 proteins in 54,219 UK-based participants (11), and the deCODE study, which includes information on 4,907 plasma proteins from 35,559 Icelandic participants (12). For analyses requiring For analyses requiring Linkage disequilibrium (LD) estimates (TWAS-FUSION, MAGMA, and MBAT-combo) we used a combined European and Asian reference panel from the 1000 Genomes Project Phase 3.

### 2.3 Statistical analysis

We performed the GWAS meta-analysis across the three datasets using METAL under its default fixed-effect model (13), a tool designed for integrating results from different studies (14). The extended major histocompatibility complex (MHC) region on chromosome 6 (26–35 Mb) was excluded because this region is highly gene-dense, highly polymorphic, and characterized by complex long-range linkage disequilibrium, which can confound association signals and complicate locus definition in GWAS analyses, and only single nucleotide polymorphisms (SNPs) with a minor allele frequency (MAF) > 0.001 were included. A genome-wide significance threshold of *P* < 5 × 10^-8^ was applied to determine association significance. Next, FUMA was used to functionally annotate the significant findings and define risk loci (15). Based on the built-in 1000 Genomes Project All LD reference panel, the software first identified independent lead SNPs (P < 5 × 10−8, r^2^ < 0.1), and then merged those that were located within 250 kb of each other into a single risk locus. We used a 250 kb distance threshold for merging lead SNPs because this is the default in FUMA and a commonly used parameter for GWAS locus definition. A 250 kb window roughly captures typical linkage disequilibrium block sizes, ensuring that closely neighboring association signals are grouped into one locus while minimizing the risk of erroneously merging independent loci. Gene annotation included both coding and regulatory regions, and eQTL associations were evaluated using the GTEx v8 database. Enrichment analyses were performed using FUMA’s built-in modules, including tissue-specific gene expression and biological pathway analyses. To further evaluate whether the extremely small association P-values were driven by systematic inflation or by true polygenic signal, we performed univariate LD score regression (LDSC) on the GWAS meta-analysis summary statistics. We report the genomic inflation factor (λGC), mean chi-square statistic, LDSC intercept, and attenuation ratio. The attenuation ratio was calculated as (intercept-1)/(mean χ^2^-1), with lower values indicating that a smaller proportion of test statistic inflation is attributable to confounding rather than polygenicity.

### 2.4 Ancestry-specific fine-mapping with SuSiEx

SuSiEx integrates summary statistics from different populations, while modeling their unique allele frequencies and LD structures. Therefore, it was used in this study to further identify potential causal variants and explore ancestry-specific genetic signals (16). We applied SuSiEx to 132 lead SNPs identified in our initial meta-analysis. For each lead SNP, we extracted a ±500 kb genomic window and conducted ancestry-specific fine-mapping. Variants with a posterior probability (POST-HOC_PROB) > 0.8 were considered to have ancestry specificity.

### 2.5 Trans-ethnic genetic correlation analysis

We used Popcorn software (Ver. 16) to analyze the trans-ethnic genetic correlation between European and East Asian populations. Both the effect correlation (*ρge*) and the impact correlation (*ρgi*) were estimated. The method uses a weighted likelihood model, and incorporates LD reference panels that we constructed for the European and East Asian populations. Detailed information on the methodology can be found in the original publication (17).

### 2.6 Proteome-wide association study

We performed a PWAS to investigate how genetic variants might influence GDM risk through protein levels. Bayesian Likelihood Integrating Summary Statistics (BLISS) was used for this analysis. It is a novel approach that builds models to impute protein expression levels based on summary-level protein quantitative trait loci (pQTL) data. For this study, we used extensive European PWAS models generated from large-scale pQTL data, including the UKB, deCODE, and ARIC studies (18). The trans-ethnic PWAS was performed using RStudio (ver. 4.4.0). Proteins with an False discovery rate (FDR)-adjusted p < 0.05 were identified as significant risk factors, indicating their potential roles in the pathophysiology of GDM. Notably, Because the protein prediction models used in BLISS were trained primarily in European pQTL cohorts (including UKB, deCODE, and ARIC), their predictive performance is expected to be higher in the European GWAS than in the East Asian GWAS (19). Differences in allele frequencies, linkage disequilibrium patterns, and potentially ancestry-specific pQTL architecture may reduce cross-ancestry portability. Therefore, differences in the number of significant PWAS signals between populations should be interpreted cautiously and may reflect both biological heterogeneity and methodological differences in model transferability and statistical power.

### 2.7 Candidate gene prioritization

We performed eQTL mapping to link genetic variants to their effects on gene expression, which helps elucidate the genetic architecture of disease susceptibility (20, 21).This analysis was performed on FUMA online platform, using transcriptomic data from 47 tissues in the GTEx dataset (excluding testis and prostate). An FDR <0.05 was applied to determine statistical significance.

We then conducted transcriptome-wide association studies (TWASs), a powerful tool for bridging the gap between genotypes and phenotypes (22). It offers deep insights by integrating the effects of multiple eQTLs into a single, robust predictor of gene expression, which is then tested for association with the disease (23). We used FUSION software to perform this analysis based on a Bonferroni-corrected *p* < 0.05.

We also used the GCTA-mBAT-combo method, which combines test statistics from multiple SNPs in a region to assess a gene’s overall association with a trait. It integrates mBAT and fastBAT statistics through a Cauchy combination method (24–26). This makes it particularly powerful in scenarios when a complex trait is collectively influenced by multiple SNPs within a gene (25). Statistical significance was set at a Bonferroni-corrected p <0.05.

We also used MAGMA (Ver. 1.08), a gene analysis tool based on a multivariable regression model (27). It aggregate SNP-level association statistics into gene-level scores to quantify the association between each gene and phenotype (28, 29). The analysis was run with default parameters, and statistical significance was set at a Bonferroni-corrected p <0.05 (30).

To prioritize causal genes using an integrative approach, we employed the Polygenic Priority Score (PoPS) method. PoPS predicts polygenic associations by integrating multiple data categories, including gene expression profiles, protein–protein interaction networks, and pathway databases (31). The gene-level association statistics and correlations were computed with MAGMA (30), and then used to fit a joint model and generate a final priority score for each gene.

Colocalization analysis was conducted to evaluate whether the genetic signals for gene expression and GDM were driven by the same causal variant (32). By applying the “coloc” package in R (Ver. 4.4.0), we estimate the posterior probabilities (PPs) for five hypotheses: (1) H0: no association with either gene expression or phenotype; (2) H1: associated with gene expression only; (3) H2: associated with phenotype only; (4) H3: associated with both traits through distinct causal SNPs; (5) H4: associated with both traits through a shared causal SNP. Following commonly used criteria, we considered colocalization evidence to be significant when PPH3 + PPH4 > 0.8 (32). We used the default settings of the coloc.abf function, with prior probabilities of 1×10^−4^ for both H1 and H2, and 1×10^−5^ for H4. We chose these prior probabilities as they are standard defaults in colocalization analyses, implying that only a very small fraction of variants are expected to be associated with each trait (~1 × 10^−4) and an even smaller fraction with both traits (~1 × 10^−5). These values are supported by empirical evidence and help maintain stringent criteria for declaring a shared causal variant.

Finally, genes proximal to the genome-wide significant loci from the GWAS meta-analysis were regarded as potential susceptibility genes. For auxiliary candidate gene prioritization, we defined the nearest gene to each lead SNP as the representative candidate gene.

### 2.8 MAGMA-based gene set and tissue expression enrichment analysis

To explore the biological pathways and tissue specificity underlying the GDM’s genetic architecture, we performed the following analyses using the MAGMA software (Ver. 1.08) (30).

First, we mapped SNP-level summary statistics to protein-coding genes based on their physical position. Gene-level association statistics were then calculated using MAGMA’s SNP-wise mean model, which aggregates multiple SNP signals within each gene while accounting for local LD patterns.

Next, gene set enrichment was performed by regressing gene-level test statistics on binary gene set membership, correcting for confounders such as gene size, gene density, and LD structure. We tested gene sets sourced from major databases, including Gene Ontology Biological Processes (GO-BP), KEGG, and Reactome. For statistical evaluation, we applied a dual threshold: Bonferroni-corrected p value (BF) < 0.2 was considered statistically significant, whereas nominal p < 0.05 was regarded as suggestive evidence of association.

Finally, we further assessed tissue-specific enrichment using MAGMA’s gene-property analysis, leveraging RNA expression profiles from GTEx v8, which includes transcriptomic data across 54 normal human tissues. For each tissue, the average gene expression level was tested for correlation with GWAS-derived gene-level Z-scores via linear regression. This approach identifies tissues in which genes with higher expression tend to have stronger GWAS associations. All analyses were adjusted for known covariates, and results were interpreted based on regression coefficients (BETA), standard errors (SE), *P*-values, and Bayes Factors.

### 2.9 Gene set polygenicity with GSA-MiXeR

To investigate whether specific biological pathways contribute disproportionally to the GDM risk, we applied GSA-MiXeR (Genome-wide Summary-based Association Mixture Enrichment Regression), a Bayesian framework that quantifies gene set enrichment based on SNP-level polygenicity and heritability.

GSA-MiXeR builds upon the MiXeR model, which estimates the overall polygenicity (total number of trait-influencing variants) and discoverability (their average effect size) from GWAS summary statistics (33). For gene set enrichment, GSA-MiXeR compares a two-component mixture model between SNPs annotated to a given gene set and genome-wide background SNPs, estimating the relative enrichment in polygenicity and heritability for each gene set.

This study utilized European GDM GWAS meta-analysis data, with a primary focus on detecting enrichment of GO-BP terms. Fold enrichment estimates computed by GSA-MiXeR were annotated to analyze the number of relevant gene sets, fold enrichment, and gene count. Finally, these gene sets were mapped to associated pathways to examine functional enrichment.

### 2.10 Genetically informed spatial mapping analysis

To understand the spatial distribution of GDM risk genes during embryonic development, we performed gsMap analysis by projecting GWAS-prioritized genes onto a reference spatial transcriptomic atlas of E16.5 mouse embryos, using the MOSTA (Mouse Organogenesis Spatiotemporal Transcriptomic Atlas) framework (34).

GWAS-significant loci were first mapped to protein-coding genes using a ±10 kb window around gene bodies. These genes were then projected onto spatial gene score maps (GS maps), which quantify the regional transcriptional activity of each gene across annotated tissue compartments based on spatial transcriptomic profiles. For each gene, its Median Gene Score Summary (GSS) and Pearson correlation coefficient (PCC) with each tissue region were calculated to assess expression intensity and tissue specificity.

To evaluate overall trait-level enrichment, we integrated all GWAS-prioritized genes into a composite GS map. The statistical enrichment of each embryonic region was quantified by comparing observed expression patterns with permuted background distributions, and visualized as –log_10_(P) values on embryo-wide spatial heatmaps. Regions with high –log_10_(P) values represent spatial hotspots of disease-relevant gene activity.

Analyses were conducted using the MOSTA web platform (https://db.cngb.org/stomics/mosta/), and tissue-level annotations (e.g., lung, liver, GI tract) were based on manual histological segmentation within the E16.5 developmental stage. We acknowledge that using a mouse embryonic atlas (E16.5) as a proxy for human pregnancy is an approximation. While developmental pathways are often conserved between mouse and human (providing a useful model), there are species-specific and temporal differences; E16.5 in mouse does not perfectly correspond to an equivalent gestational time in humans. Therefore, the gsMap results are treated as exploratory, and we interpret them cautiously given the limitations of cross-species extrapolation.

## 3 Results

### 3.1 GWAS meta-analysis

**Figure 2:**
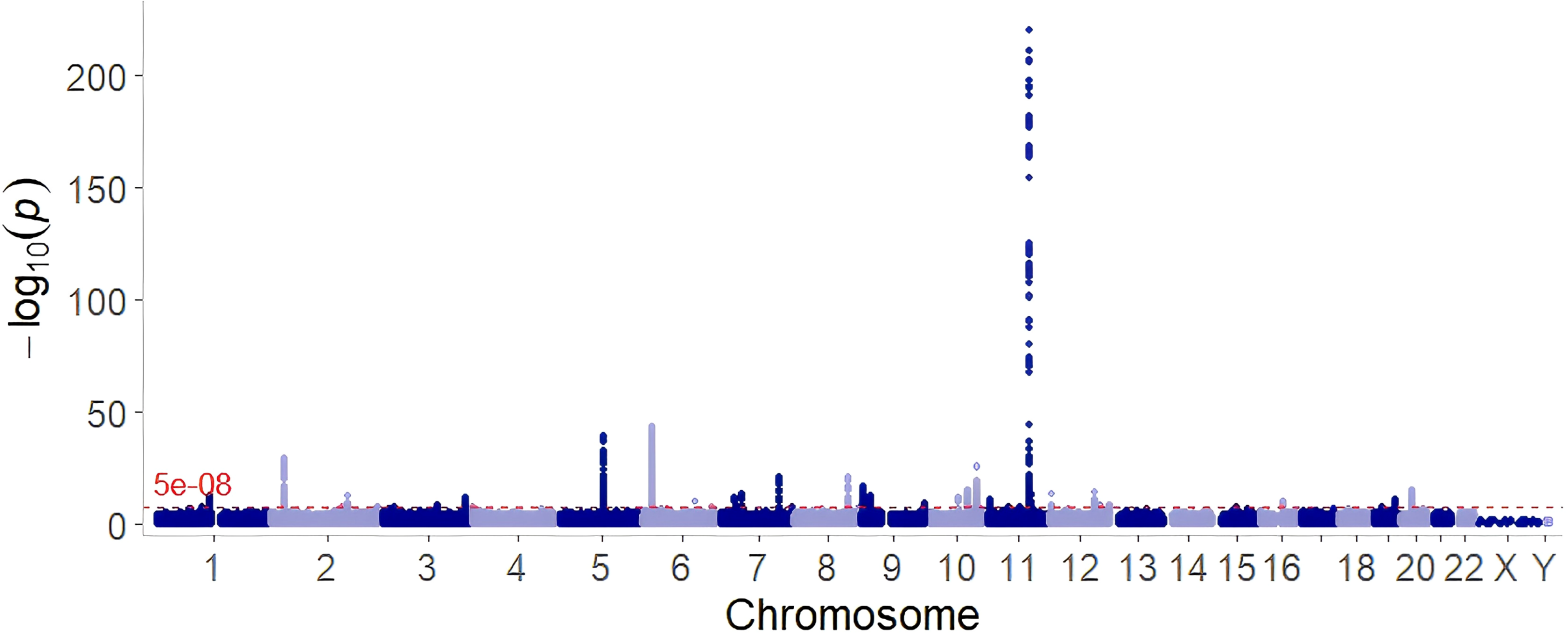
Manhattan plot of the GWAS meta-analysis for GDM.

To systematically identify genetic risk factors for GDM across different populations, we integrated GWAS data from three large cohorts and conducted a meta-analysis (**Figure 2**). After excluding variants with a MAF ≤ 0.001 and removing the 26–35 Mb region of chromosome 6, our analysis identified a total of 37 independent loci, containing 132 GDM-associated lead SNPs, using FUMA (**Supplementary Table 1**). The strongest association signal was rs10830963 on chromosome 11, which is located near the *MTNR1B* gene, with a P-value < 5 × 10^−308^ and a well-established susceptibility locus for both GDM and T2D (35, 36). Several other key variants closely correlated to glucose metabolism and pancreatic β-cell function were also identified, including rs3847554 (Chr11, *SNRPGP16*, P = 4.85 × 10^-180^), rs7766070 (Chr6, *CDKAL1*, P = 3.18 × 10^-44^), rs12332295 (Chr5, *CTD-2337A12.1*, P = 1.06 × 10^-39^), and rs11020132 (Chr11, *MTNR1B*, P = 1.86 × 10^-30^) (37, 38). These loci were supported by robust statistical significance, with some showing strong overlap with known T2D-associated variants, suggesting that GDM shares a common genetic basis with other glucose-related metabolic disorders. In addition to replicating known signals, our analysis also uncovered less frequently reported loci, such as rs6048205 (Chr20, *LINC00261*, P = 4.481 × 10^-16^). To contextualize the extremely small P-values observed in the GWAS meta-analysis, we assessed genomic inflation using LDSC. The genomic inflation factor was 1.1183, the mean chi-square statistic was 1.151, and the LDSC intercept was 1.0095 (SE = 0.0065). The attenuation ratio was 0.063 (SE = 0.043), indicating that only a small proportion of the observed inflation was attributable to confounding, whereas most of the inflation was likely due to polygenicity and large sample size.

### 3.2 Functional annotation with FUMA

**Figure 3:**
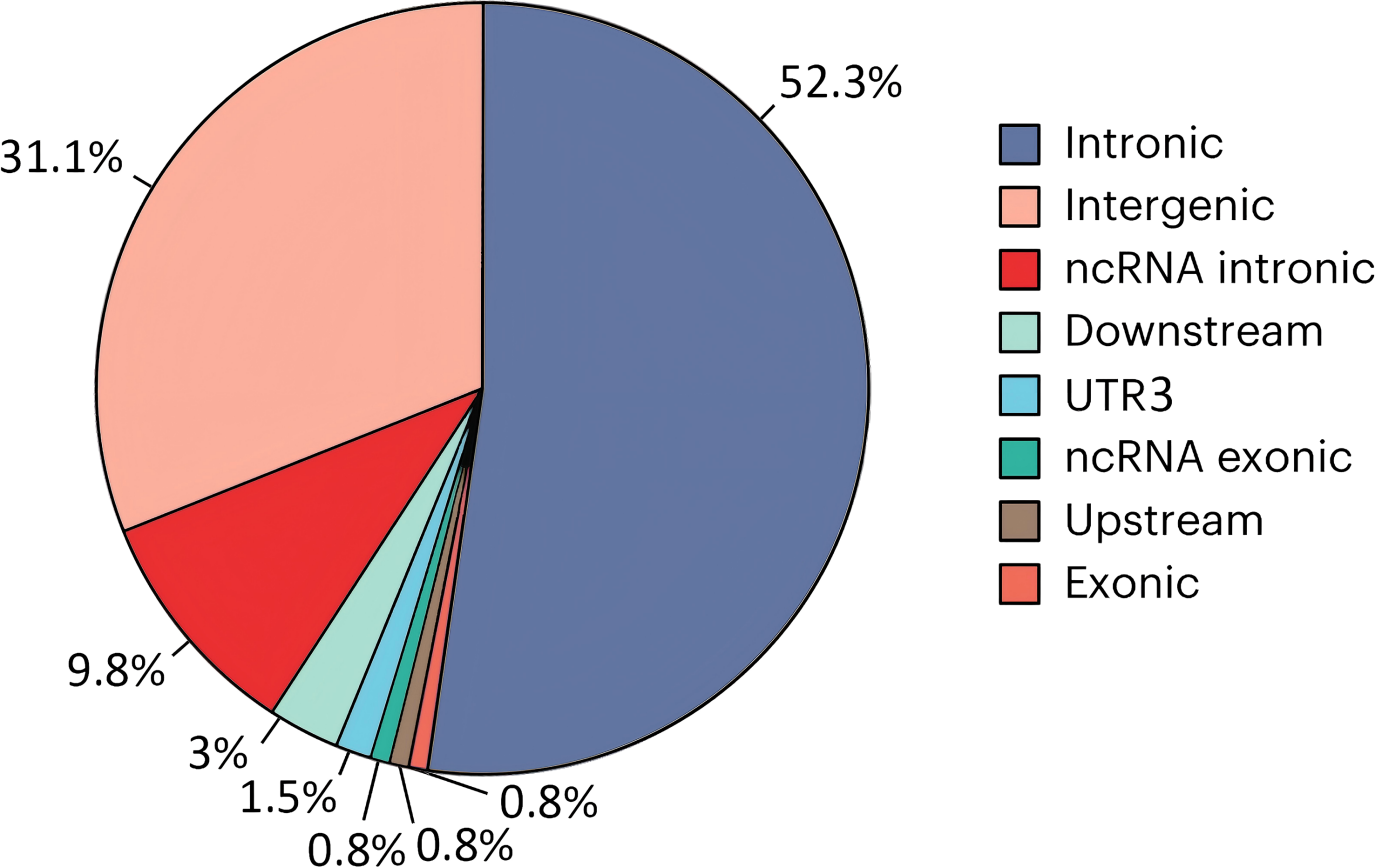
Functional distribution of lead SNPs identified in the GWAS meta-analysis. This pie chart shows the proportion of the total 132 lead SNPs that fall within intronic, intergenic, exonic, or other genomic regions based on FUMA annotations. Percentages are indicated out of each sector.

To further explore the biological functions of the 132 lead SNPs identified in our GWAS, we performed a comprehensive functional annotation using the FUMA platform. Our analysis revealed several insights into how these genetic variants might contribute to GDM risk. First, the majority of variants were non-coding. As shown in **Figure 3**, 52.3% (69 SNPs) were located in intronic regions and 31.1% (41 SNPs) were in intergenic regions. Only one variant (rs9350269, a missense variant in the *CDKAL1* gene) was located in a protein-coding exon. Second, a significant number of SNPs showed signs of regulatory function. A total of 42 lead SNPs colocalized with eQTLs (eqtlMapFilt = 1), and five SNPs had high RegulomeDB score (1 or 2), suggesting potential cis-regulatory effects. Interestingly, while the exonic variant in *CDKAL1* had a low CADD score (8.246), indicating limited pathogenic potential, some non-coding variants—such as an intronic SNP in the *FAM46C* locus—showed higher CADD scores (up to 13.23), suggesting potential functional relevance. Through positional mapping, 92 SNPs were mapped to nearby genes within a 10 kb window (**Supplementary Table 1**); however, no significant chromatin interaction signals were detected for these loci. Taken together, these findings suggest that GDM-associated variants may influence disease risk by acting as regulatory elements that modulate gene expression, rather than by directly altering protein structures.

### 3.3 Identification of ancestry-specific and shared loci with SuSiEx

**Figure 4:**
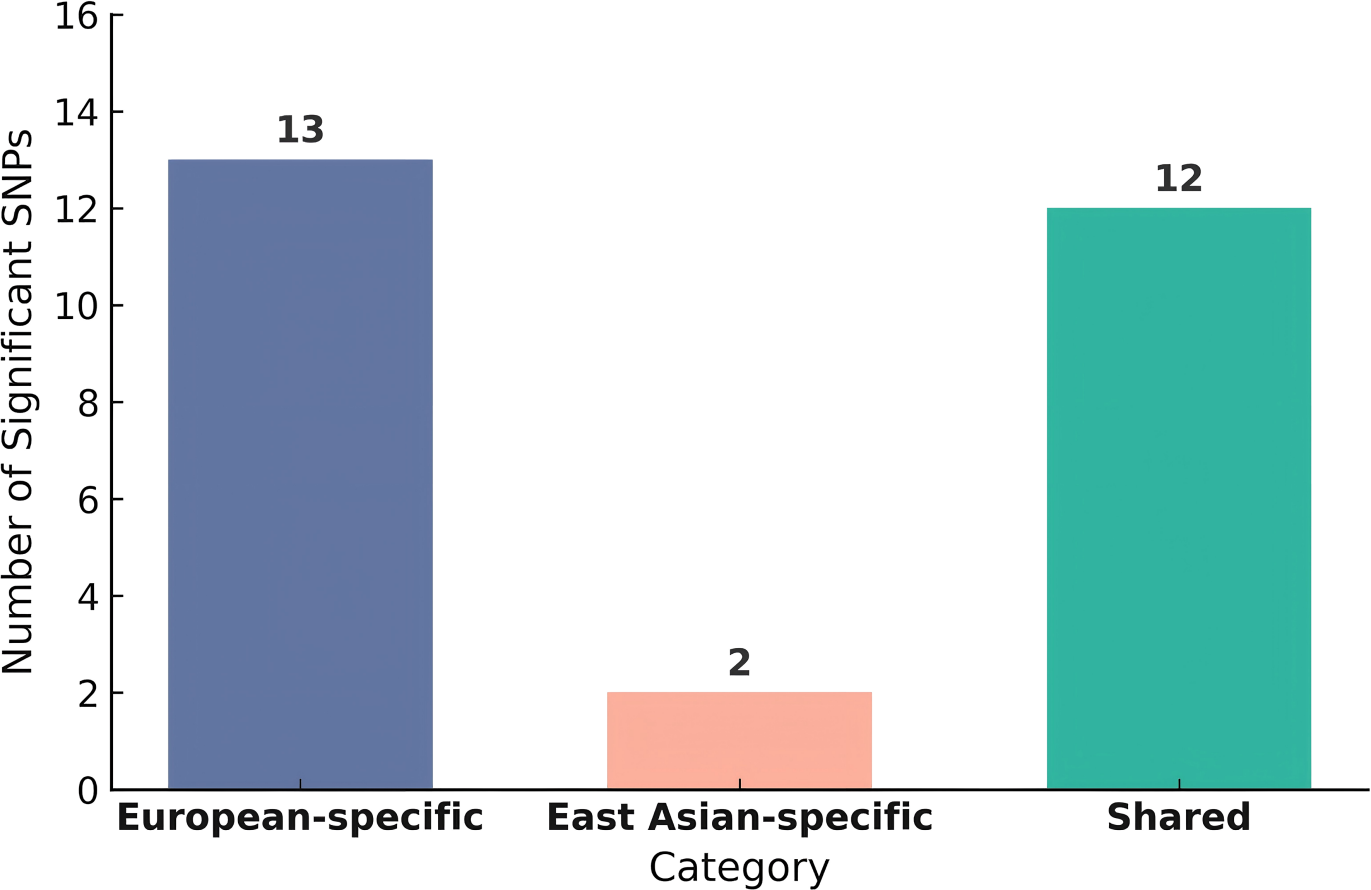
Distribution of ancestry-specific loci identified by SuSiEx. The bar plot shows the number of significant loci that are specific to European ancestry, specific to East Asian ancestry, or shared between both ancestries.

A total of 27 ancestry-specific loci were identified through SuSiEx analysis. We dissect these signals into three distinct categories based on ancestry, as shown in **Figure 4** (**Supplementary Table 2**). These include: 13 European-specific SNPs, including rs28624681, rs10860207 and rs2488087, which showed a significant association only in the European population; 2 East Asian-specific SNPs, including rs61160304 and rs116904139, which were significant only in the East Asian population; and 12 shared SNPs, including rs2394529; rs10882101 and rs2901609, which were significantly associated with GDM in both populations. These ancestry-specific or cross-population shared loci may provide a strong foundation for future mechanistic studies, the development of more accurate, ancestry-aware risk prediction models, and targeted efforts for functional validation.

### 3.4 Shared genetic architecture across ancestries assessed by popcorn

Our Popcorn analysis assessed the genetic correlation of GDM between the European and East Asian populations, revealing the SNP-based heritability and the degree of genetic overlap between them. The analysis estimated the heritability of GDM to be 0.0210 (SE = 0.0031, P = 7.79×10^-12^) in the European population (h_1_^2^) and 0.0624 (SE = 0.0102, P = 1.11×10^-9^) in the East Asian population (h_2_^2^). We found a strong and significant positive correlation between the two populations in both the direction of SNP effects(ρge = 0.596, SE = 0.102, P = 7.47×10^-5^) and the magnitude of these effect (ρgi = 0.632, SE = 0.111, P = 8.94×10^-4^). These findings indicate that GDM has a significant genetic correlation and consistent genetic effect profile between European and East Asian populations. Despite differences in allele frequencies and environmental exposures, most GDM-associated risk loci demonstrated a consistent direction of effect, suggesting a highly shared genetic basis for the disease across ancestries. Meanwhile, these results also support the importance of conducting ancestry-specific studies to identify population-specific risk loci that also act as a GDM contributor.

### 3.5 PWAS analysis

Our PWAS investigated how genetically-predicted protein levels are associated with GDM risk in both European and East Asian populations. In the European population, the analysis identified 59 genes whose predicted protein levels were significantly associated with GDM risk (FDR-adjusted *p* < 0.05). In the East Asian population, two genes met the same significance threshold (FDR-adjusted *p* < 0.05). Notably, a consistent protein association signal was observed across both populations for the *ATRAID* protein (**ST 3**). In both Europeans and East Asians, higher *ATRAID* levels acted as a risk factor for GDM (*β* > 0). These findings suggest that *ATRAID* may represent a cross-ancestry, shared pathogenic factor for GDM. The marked difference in the number of significant PWAS hits between Europeans (59 proteins) and East Asians (2 proteins) should not be interpreted solely as evidence of stronger protein-level genetic effects in Europeans. First, the BLISS protein imputation models were trained mainly in European cohorts, which may favor prediction accuracy in the European GWAS. Second, the European GDM datasets in our study had a larger overall sample size than the East Asian dataset, providing greater power to detect associations. Third, ancestry differences in allele frequencies and LD patterns, as well as possible population-specific pQTL architecture, may further reduce the portability of protein prediction models to East Asian samples. Nevertheless, the shared association of *ATRAID* across both populations suggests that at least part of the protein-mediated genetic architecture of GDM is shared across ancestries.

### 3.6 Prioritization of causal genes

To determine the most significant causal genes for GDM, we performed a gene prioritization process that integrated evidence from six different analyses.

**Figure 5:**
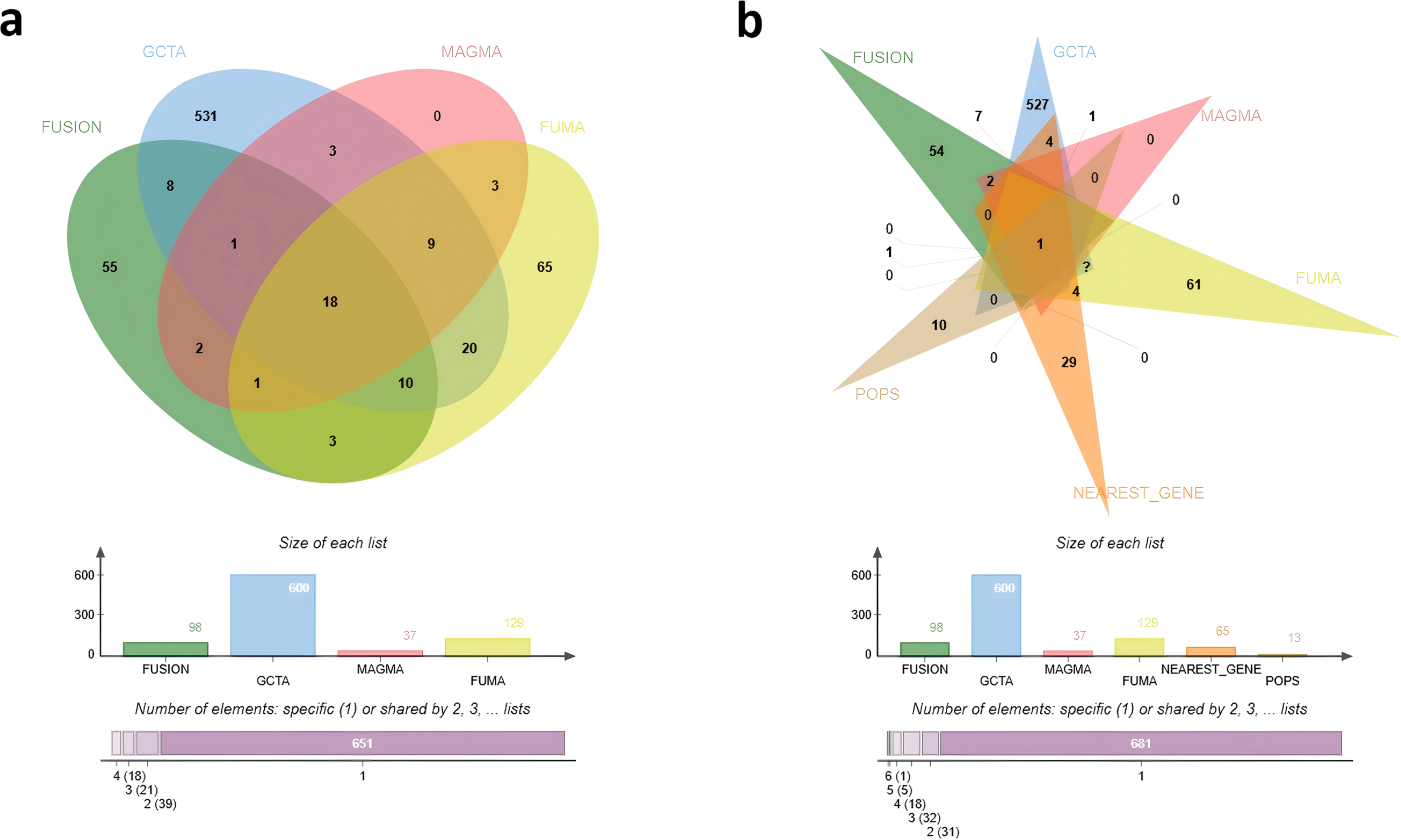
Overlap of prioritized GDM candidate genes identified by multiple methods. The numbers in each section of the diagrams represent the count of identified genes. **(a)** This Venn diagram shows the overlap of candidate genes identified by the initial four methods: *FUMA*, *FUSION*, *MAGMA*, and *GCTA*. **(b)** This Venn diagram identified illustrates the overlap of genes identified by all six methods, adding PoPS, and nearest gene approach to the initial four.

We first integrated evidence from four powerful analytical approaches: eQTL mapping, which provides a comprehensive understanding of the genetic basis underlying phenotypic variation; MAGMA, which enables gene-centric analysis based on protein-coding gene data; TWAS-FUSION, which facilitates the identification of candidate genes associated with complex traits; GCTA-mBAT-combo, which is particularly useful for detecting pleiotropy. The initial convergence of evidence identified 18 high-confidence genes, including *ELL2*, *IGF2BP2*, *GCKR*, *NRBP1*, and *PCSK1*, among others (**Figure 5a**; **Supplementary Table 4**). The 18 high-confidence genes represent the intersection supported simultaneously by four approaches: eQTL mapping, MAGMA gene-level association, TWAS-FUSION, and GCTA-mBAT-combo.

Subsequently, we applied two additional methods, PoPS and the nearest gene approach, to further prioritize candidate genes from the above identified genes. PoPS improves the gene prioritization accuracy within GWAS loci, providing valuable insights into the complex interactions, while the nearest gene method consider genes located closest to significant variants. After analyzing the intersection of all methods, only one candidate gene, *ELL2*, met these stringent criteria (**Figure 5b**; **Supplementary Table 5**).

Finally, to investigate if the GWAS and QTL signals were driven by the same causal variants, we conducted colocalization analysis (32). The results showed co-existing eQTL and GWAS signals for 18 genes (PP.H3 + PP.H4 >0.80), including *ELL2*, *NRBP1*, *PCSK1* and *SMCO4*. The signals for our top candidate, *ELL2*, were observed in both esophageal mucosa and thyroid tissue in relation to GDM. However, the posterior probability analysis suggested that the eQTL and GWAS signals are driven by distinct causal variants (PP.H3 + PP.H4 > 0.9997 and 0.9408, respectively); as reflected by a PP.H4 < 0.01 (no strong evidence for a shared causal variant). This suggests that the eQTL and GWAS associations at the *ELL2* locus are likely due to different proximal causal variants rather than a single shared variant.

### 3.7 MAGMA pathway and tissue enrichment analysis

Our MAGMA analysis tested 16,994 gene sets and identified multiple biological pathways enriched with GDM-associated genetic signals. We identified two GO terms that met Bonferroni-corrected significance, including: regulation of insulin secretion (*P* = 8.75× 10^-6^; BF = 0.149), which suggests a central role of impaired insulin regulation in GDM; and regulation of protein secretion pathway (*P* = 8.01 × 10^-6^; BF = 0.136), which highlights a broader involvement of secretory machinery in glucose metabolism.

These findings provide functional support for the relevance of pancreatic β-cell activity and protein trafficking processes in the development of GDM.

Although no tissues reached statistical significance after multiple-testing correction, nominal associations were observed between tissue-specific gene expression and GDM-associated genetic signals. The strongest correlation was detected in the esophagus gastroesophageal junction (BETA = 0.0352, P = 0.0045, BF = 0.24), followed by the uterus (BETA = 0.0284, P = 0.0067, BF = 0.36).

### 3.8 GSA-MiXeR prioritization of key gene sets

To prioritize key gene sets, we used GSA-MiXeR to identify and rank pathways that showed nominal significance in the MAGMA analysis (Bonferroni-corrected p<0.05). Gene sets with positive AIC values in GSA-MiXeR were considered. For GDM, GSA-MiXeR prioritized the “negative regulation of hexokinase activity” gene set from among the nominally significant MAGMA pathways. This set contains five genes (*COX11*, *FOXA2*, *GCKR*, *MIDN*, and *PRKN*) and showed a 1.70-fold enrichment.

We then performed exclusion analysis to find the most influential gene within this pathway. When *FOXA2* was removed from the set, the enrichment score dropped from 1.70 to 0.73, suggesting that *FOXA2* is the primary contributor of this pathway’s significant association with GDM. *FOXA2* is notable as a transcription factor expressed in multiple metabolic tissues (e.g., liver and pancreas) and crucial for insulin secretion and metabolic homeostasis. Its dominant contribution to this pathway suggests that *FOXA2* could be a key mediator, potentially linking GDM to alterations in maternal liver function or pancreatic β-cell activity, as well as to developmental processes in endoderm-derived organs.

### 3.9 Genetic spatial mapping result

**Figure 6:**
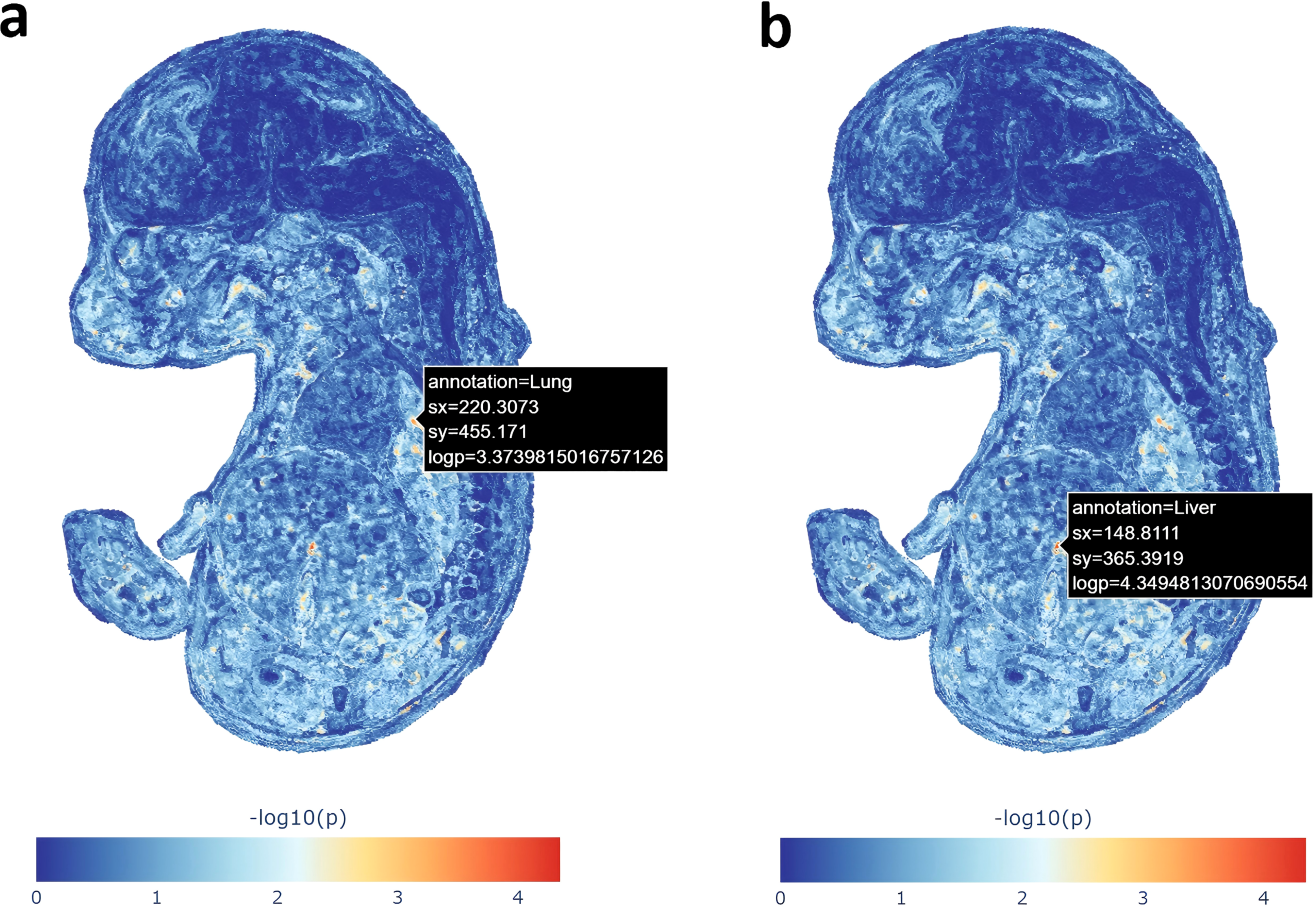
Genetic spatial mapping of GDM risk genes in mouse embryo. The color scale on the heatmaps represents the statistical significance of enrichment, measured as –log_10_(P-value). **(a)** The spatial enrichment of GDM risk genes in the fetal lung. **(b)** The spatial enrichment of GDM risk genes in the fetal liver.

Our genetic spatial mapping analysis projected GDM risk genes onto transcriptomic atlas of a developing mouse embryo to identify the most active hotspots for these genes. Notably, two regions demonstrated significant spatial enrichment: lung (*–log_10_(P)* = 3.37) and liver (*–log_10_(P)* = 4.35) (**Figure 6**). These results show that fetal pulmonary and hepatic tissues may represent the most intense sites of activity for the GDM susceptibility genes during embryonic development. These findings support the hypothesis that the GDM risk may partially originate from transcriptional disturbances during the formation of endoderm-derived metabolic and respiratory organs.

## 4 Discussion

We conducted a large-scale cross-ancestry GWAS meta-analysis for GDM, involving 31,469 cases and 338,305 controls. The analysis has made some significant discoveries regarding the disease’s genetic architecture. First, our meta-analysis identified *ELL2* and *ATRAID* as novel genetic risk factors for GDM, neither of which has been previously implicated in GDM or related metabolic disorders. Second, our results underscore that impaired insulin secretion is a central pathway in GDM. This was supported by MAGMA analysis, which identified significant over-representation of GDM-associated genes in pathways regulating insulin and protein secretion. Third, our GSA-MiXeR analysis identified the *“negative regulation of hexokinase activity”* as the top-ranked pathway, a signal largely driven by *FOXA2*. Finally, in a novel application of spatial transcriptomics, we found that GDM risk genes are significantly enriched in the developing fetal lung and liver.

*ELL2* is primarily known for its role as an RNA polymerase II elongation factor in processes such as immunoglobulin production, and its link to multiple myeloma risk (39) and prostate cancer (40). In our study, *ELL2* emerged as a candidate GDM risk gene supported by multiple lines of evidence, including eQTL signals in esophageal and thyroid tissues. However, our colocalization analysis suggested that the genetic signals for *ELL2*’s expression and GDM risk, while closely located, seem to be driven by distinct causal variants, indicating that the genetic regulation and disease risk may operate independently. Beyond its canonical role as an RNA polymerase II elongation factor, *ELL2* functions within the super elongation complex (SEC) to promote productive transcription by limiting promoter-proximal pausing and increasing elongation efficiency (41, 42). Notably, *ELL2* has been best characterized in highly secretory plasma cells, where it reshapes RNA processing programs to support immunoglobulin secretion (43), underscoring a potential capacity of *ELL2* to influence transcriptional output under conditions of high biosynthetic demand. From a genetic perspective, noncoding risk variants at the *ELL2* locus have been shown to modulate *ELL2* expression in human disease contexts (44), supporting the plausibility that regulatory variation could alter *ELL2* dosage.

Although direct evidence linking *ELL2* to glucose homeostasis in pancreatic β-cells or hepatocytes is currently lacking, transcription elongation control via P-TEFb/CDK9 and related elongation machinery has been implicated in the induction of metabolic gene programs in insulin-sensitivity–relevant tissues (45). Given that pregnancy requires robust β-cell functional adaptation and coordinated hepatic glucose production to maintain maternal–fetal glucose balance (46), we speculate that altered *ELL2*-mediated elongation or RNA-processing dynamics could influence the capacity of β-cells to mount adaptive transcriptional responses or affect hepatic transcriptional programs relevant to gluconeogenesis during pregnancy. These hypotheses remain to be tested, and targeted functional studies in β-cells/hepatocytes (e.g., perturbation of *ELL2* expression followed by insulin secretion and gluconeogenic flux assays) will be necessary to establish causality. This is the first study to associate *ELL2* with dysglycemia, providing a new perspective on GDM genetics beyond classical metabolic genes. The second novel locus, *ATRAID*, also has no prior GDM association. It’s a membrane-associated protein known to participate in cell cycle arrest, apoptosis, and bone metabolism (47, 48). Its broader functions in apoptosis, cell proliferation, and differentiation—as evidenced by its regulation of cyclin D1 and osteoblast differentiation—suggest possible mechanisms in how pancreatic β-cells adapt to the metabolic stress during pregnancy (49). Notably, emerging functional data show that silencing the *ATRAID* gene in pancreatic islet cells leads to increase insulin secretion under high-glucose conditions, suggesting it normally acts as a negative regulator of insulin release (50). Another recent PubMed-indexed study showed that siRNA-mediated knockdown of Atraid in INS-1 832/13 β-cells increased Ins1/Ins2 expression and enhanced insulin secretion under high-glucose conditions, suggesting that *ATRAID* may normally restrain β-cell secretory output (51). This aligns with our cross-ancestry PWAS finding that higher genetically predicted *ATRAID* levels are associated with increased GDM risk. During normal pregnancy, maternal β-cells must mount a coordinated adaptive response—characterized by augmented insulin synthesis and secretion, enhanced intercellular coupling, and a lower glucose threshold for secretion—partly mediated through pregnancy-associated hormonal cues and downstream signaling that reshape β-cell stimulus–secretion coupling, including calcium dynamics (52). Emerging human data further highlight that pregnancy is associated with measurable islet remodeling and altered expression of key receptors involved in gestational β-cell adaptation (53). If *ATRAID* expression or activity is abnormally high in maternal islets, it could blunt the compensatory increase in insulin release required in late gestation, thereby contributing to hyperglycemia. In other words, dysregulation of *ATRAID* might impair the physiological adaptation of β-cells to pregnancy-induced insulin resistance. Further experimental work is needed to examine how *ATRAID* is regulated during gestation and to confirm its role in β-cell function under the metabolic stress of pregnancy.

Our MAGMA analysis confirmed that impaired insulin secretion is a notable genetic occurrence in GDM. We found a significant enrichment of GDM-associated signals in the GO term *“regulation of insulin secretion”* (P = 8.75×10^-6^). This dovetails with abundant prior evidence, as many of our top GDM loci – including well-known T2D genes such as *MTNR1B*, *TCF7L2*, *CDKAL1* and *GCKR* – are related to β-cell function, supporting the notion that GDM shares a core genetic architecture with T2D (8, 54). Notably, *TCF7L2*, the strongest T2D locus, has also been linked to GDM risk via its effect on insulin secretion (55). These findings implicate pancreatic islet dysfunction as a key genetic and pathophysiological theme in GDM, where genetic variants reduce insulin secretion capacity and precipitate hyperglycemia during the metabolic stress of pregnancy. Our pathway analysis also pinpointed broader secretory and metabolic processes. The *“regulation of protein secretion”* pathway was significantly enriched in our MAGMA results (P = 8.01×10^-6^), highlighting the involvement of the general secretory machinery in glucose homeostasis and possibly GDM. This could reflect challenges such as endoplasmic reticulum stress or secretory pathway demands in maternal tissues and placenta during GDM.

A striking result from our GSA-MiXeR analysis was the top ranking of the *“negative regulation of hexokinase activity”* pathway. This signal was largely driven by FOXA2, which is essential for glucose responsiveness in both pancreatic β-cells and the liver (56). The enrichment of this pathway reinforces that genetic perturbations in glucose handling (in liver and pancreas) induce GDM susceptibility. FOXA2 is known to play a role in cell identity maintenance and metabolic gene regulation (56). Animal studies demonstrate that its deletion in β-cells results in severe hypoglycemia and dysregulated insulin secretion, underscoring its essential role in postnatal β-cell function (57). Emerging human and genetic data support FOXA2’s involvement in diabetes susceptibility. Furthermore, human genetic data support FOXA2’s involvement in diabetes susceptibility (58). While our MAGMA tissue enrichment analysis did not yield any tissue passing strict significance threshold, we observed nominal associations pointing to the involvement of insulin target tissues. Genes highly expressed in subcutaneous and visceral adipose tissue tended to show enrichment in GDM loci (top MAGMA z-scores for adipose). Adipose tissue is central to insulin resistance, and maternal obesity/insulin resistance is a well-known contributor to GDM (59).

One novel aspect of our study was the use of gsMap to project GDM genes onto a developing mouse embryo. Intriguingly, by leveraging the gsMap at embryonic day 16.5 (E16.5), we found a significant enrichment of GDM risk genes in the developing fetal lung (–log_10_P = 3.37) and liver (–log_10_P = 4.35). One possibility is that this reflects true developmental programming effects: maternal hyperglycemia and genetic factors in GDM might influence the development or gene expression patterns of the fetus’s lung and liver, potentially predisposing the offspring to metabolic or respiratory complications later in life (60, 61). This interpretation is consistent with the developmental origins of health and disease (DOHaD) framework, wherein an adverse intrauterine environment can induce lasting changes in fetal organ structure and function. Another possibility is that the fetal lung and liver enrichment indicates shared transcriptional networks between these fetal organs and the mother’s metabolic organs. In other words, many GDM-related genes might be highly expressed in both fetal lung/liver and adult tissues like the pancreas or liver, so the spatial “hotspots” could be highlighting common pathways rather than direct fetal effects. A third explanation is that the result could partly arise from the specifics of the mouse model — for example, certain tissues (like lung and liver) may show higher gene activity at E16.5 in mice, and this might not fully translate to human pregnancy. This observation prompts a new hypothesis that GDM risk may be partly programmed by disturbances during the development of these key fetal organs. The liver and lung are both critical endoderm-derived organs that mature rapidly in late gestation (62). The liver is the main site of fetal glucose storage (glycogen synthesis), and the maturation of fetal lung (surfactant production) is hormone-sensitive and often delayed in diabetic pregnancies (63). It is important to note that our spatial analysis was conducted using a mouse embryo atlas as a proxy; thus, careful validation in human tissues (e.g. placenta, fetal organs) is further needed. Interestingly, while the mouse *placenta* was not highlighted in our analysis (it was not part of the embryonic atlas used), other human studies do suggest the placenta’s role in GDM. The recent FinnGen GDM GWAS reported that several GDM-specific loci map to genes with placenta-specific expression or to pregnancy hormones (54). For instance, GDM is known to impede fetal lung maturation via excess fetal insulin and placental hormones, leading to neonatal respiratory distress (64).

Accumulating evidence indicates that GDM is associated with subtle maternal myocardial alterations that may not be apparent on conventional echocardiography but can be sensitively detected using speckle tracking echocardiography (STE). Several prospective and observational studies have consistently demonstrated that women with GDM exhibit impaired left ventricular global longitudinal strain (GLS), despite preserved left ventricular ejection fraction and normal chamber dimensions, supporting the presence of subclinical myocardial dysfunction during pregnancy. For example, Di Corpo et al. reported significantly reduced GLS in GDM pregnancies compared with normoglycemic controls, highlighting the ability of STE to detect early myocardial deformation abnormalities that are otherwise overlooked by standard echocardiographic parameters (65). These findings have been reinforced by subsequent studies and meta-analyses, which consistently show lower LV-GLS values in women with GDM, underscoring the robustness and reproducibility of STE-derived strain abnormalities across different populations and imaging protocols (66). Additional two-dimensional STE studies further confirm that myocardial strain impairment can be detected even in well-controlled GDM, suggesting that transient gestational hyperglycemia itself may be sufficient to induce early functional myocardial changes (67). Importantly, systematic reviews have proposed STE as a promising tool not only for antenatal assessment but also for postpartum cardiovascular risk stratification in women with a history of GDM, a population known to have an increased long-term risk of cardiometabolic disease (68). Collectively, these data emphasize the clinical value of STE as a sensitive imaging biomarker for early myocardial involvement in GDM and support its potential integration with genetic and molecular risk profiling to improve cardiovascular surveillance and personalized risk assessment in affected women. Future research should investigate whether the novel susceptibility genes identified in our study, particularly *ELL2* and *ATRAID*, or the enriched pathways regarding insulin and protein secretion, contribute to the pathophysiological mechanisms underlying this subtle myocardial dysfunction in GDM patients.

Several limitations must be considered when interpreting our results. First, our meta-analysis results were derived predominantly from European and East Asian cohorts, potentially limiting their generalizability to other ancestries such as African or South Asian populations. Future studies including more diverse populations are required to validate and extend these results. Second, although our integrative annotations (e.g., MAGMA, GSA-MiXeR, gsMap) support the involvement of newly identified loci (e.g., *ELL2*, *ATRAID*), no *in vitro* or *in vivo* functional experiments were conducted to confirm their causal effects in GDM pathogenesis. Third, the gsMap enrichment analysis depended on a mouse embryonic spatial transcriptome. While this provides developmental context, interspecies and temporal differences may affect the accuracy of its extrapolation to human pregnancy. An additional limitation is that the PWAS models were derived predominantly from European pQTL resources. Therefore, the smaller number of significant PWAS signals in East Asians may partly reflect reduced cross-ancestry portability and lower statistical power, rather than the absence of biologically relevant protein-mediated effects. Future studies using ancestry-matched East Asian pQTL reference panels are needed to better clarify shared versus population-specific protein associations in GDM. While our study highlights potential biological targets, the clinical utility of these loci for risk prediction, early diagnosis, or personalized interventions has yet to be determined.

## 5 Conclusions

In summary, based on a GWAS meta-analysis of data from European and East Asian populations, we identified *ELL2* and *ATRAID* as two novel susceptibility genes whose expression is associated with the GDM risk. We also identified three crucial GDM-associated pathways: *regulation of hexokinase activity*, *regulation of insulin*, and *regulation of protein*. These findings provide new insights into the complex genetic architecture of GDM. The identification of these novel genes and pathways confirms their importance and warrants further research and clinical trials to establish their potential as therapeutic targets.

## Data Availability

Publicly available datasets were analyzed in this study. This data can be found here: GCST90566419 GCST90043951 FinnGen R12.
